# Quantifying Mechanical Properties of the Patellar and Achilles Tendons Using Ultrasound Shear Wave Elastography: A Pilot Study

**DOI:** 10.3390/diagnostics15070879

**Published:** 2025-04-01

**Authors:** William A. Berrigan, Kevin Cipriano, Kirk A. Easley, Ken Mautner

**Affiliations:** 1 Department of Orthopedics, University of California San Francisco, San Francisco, CA 94143, USA; 2 Department of Orthopedics, Emory University School of Medicine, Atlanta, GA 30322, USA; 3 Rollins School of Public Health, Emory University, Atlanta, GA 30322, USA

**Keywords:** POCUS, ultrasound, elastography, tendinopathy, tendon, orthopedics, imaging

## Abstract

(1) **Background**: Patellar and Achilles tendon injuries have become increasingly prevalent, particularly among active populations and athletes, leading to significant functional impairments. While B-Mode ultrasound has been useful in the diagnosis of these injuries, its capacity to assess tendon mechanical properties, such as stiffness, is limited. Shear wave elastography (SWE) offers a promising alternative by measuring tissue stiffness, which may enhance the evaluation of tendon health. Previous studies have established that SWE can differentiate healthy tendons from those with pathological changes. However, reference values for specific tendon types, including the patellar and Achilles tendons, remain limited. This study aims to provide preliminary baseline SWE values for these tendons in a healthy cohort. (2) **Methods**: In this cross-sectional study, healthy volunteers aged 18–65, with no history of lower extremity injury, were assessed using a Samsung RS85 Prestige ultrasound system with a 14L-2 MHz transducer. SWE measurements were obtained from the patellar tendon at a single location and from the Achilles tendon at both the midportion and insertional sites. All assessments followed a standardized protocol to ensure consistency and minimize variability. (3) **Results**: A total of 54 healthy adult participants were included. The mean SWE value for the patellar tendon was 96.3 (SD = 10.9 kPa), with males showing significantly higher stiffness than females (99.3 kPa vs. 93.8 kPa, *p* = 0.009). A higher BMI was associated with lower stiffness in the patellar tendon. The mean SWE values for the Achilles tendon were 101.7 (SD = 16.2 kPa) at the insertion and 145.6 (SD = 18.8 kPa) at the midportion. (4) **Conclusions**: This study provides SWE values for the patellar and Achilles tendons in healthy individuals, which can serve as a foundation for future research and clinical applications. These values may help in the comparison of healthy and pathological tendons, particularly in the context of tendinopathies, tendon tears, and treatment monitoring. While shear wave elasticity shows promise as a tool for diagnosing and monitoring tendon injuries and degeneration, more research is required to establish its precise reliability and validity in clinical practice.

## 1. Introduction

The patellar and Achilles tendons are subjected to significant stress during high-impact activities, making them particularly vulnerable to injury. Such tendon injuries can profoundly affect athletic performance and overall quality of life. To better manage and prevent these injuries, there is an increasing demand for an imaging technique that can accurately assess tendon pathology, identify potential issues before they lead to injury, and monitor responses to treatment. While standard brightness-mode (B-Mode) ultrasound is commonly used for tendon imaging, it has limitations—especially in its inability to measure key mechanical properties like tendon stiffness and elasticity, which are important for understanding tendon health and tracking injury progression [1,2,3].

Ultrasound shear wave elastography (SWE) has become a valuable tool in musculoskeletal imaging, offering a non-invasive way to evaluate tendon stiffness. Research has shown that SWE can reliably measure tendon stiffness over time [4], and studies have established normative stiffness values for specific tendons using this technology [5]. SWE has also proven effective in distinguishing between healthy tendons and tendinopathic tendons, which often demonstrate higher or lower stiffness values depending on the tendon due to degenerative changes, and ruptured tendons, characterized by markedly reduced stiffness or loss of elasticity [4]. Furthermore, it holds potential as a biomarker for monitoring treatment response and providing prognostic insights [5].

Despite its promise, a limitation of SWE is the variability of measurements, which can be influenced by factors such as differences in equipment, operator technique, and patient positioning. Previous studies have highlighted the inconsistent reliability of SWE measurements across different tendon types and systems, emphasizing the need for standardized protocols [6,7]. Additionally, establishing reference values for normal tendon stiffness is crucial for effectively comparing healthy and pathological tissues.

The primary aim of this feasibility study is to collect pilot data on tendon stiffness in the patellar and Achilles tendons in healthy subjects using a single ultrasound SWE system. The goal is to develop a standardized protocol for SWE in clinical practice, ultimately enabling its use in assessing tendon health and guiding treatment decisions. A secondary aim is to investigate the intra-observer reliability of SWE measurements, evaluate the variability of tendon stiffness within and between subjects, and explore how factors like sex and body mass index (BMI) might influence stiffness values. We hypothesize that tendon stiffness will show significant variability across subjects and that sex and BMI may affect stiffness measurements.

## 2. Materials and Methods

### 2.1. Study Design

This was a cross-sectional study performed at a tertiary referral center for sports medicine. Institutional Review Board approval was obtained prior to the initiation of this study.

### 2.2. Participants

Participants were included if they were healthy adults aged 18–65 without active lower extremity injury or systemic, metabolic, or endocrine disorders. The decision to recruit participants across a broad age range was to explore how tendon stiffness and elasticity might vary across different age groups given that tendon properties are known to change with age.

Participants were excluded if they had lower extremity surgery or previous trauma; orthopedic knee injuries such as tendinopathy, bursitis, ligament, and meniscus injuries; neurological or cardiopulmonary diseases; rheumatic disease such as gout, rheumatoid arthritis, and systemic lupus; active pain in the Achilles or patellar tendons; inability to consent; not met the age requirement; were pregnant; or were prisoners. B-mode ultrasound imaging was performed on all subjects prior to enrollment to confirm the absence of tendinopathy or other pathological changes in the tendons. All subjects were asked to withhold from physical activity for several hours prior to the examination.

The demographic data collected included sex, age, BMI, dominant leg, and Tegner Activity Scale (TAS) score. TAS score was used to assess the participants’ level of physical activity, ranging from sedentary to highly active individuals.

### 2.3. Shear Wave Elastography

Imaging procedures: A Samsung RS85 Prestige Ultrasound machine (Samsung Medison, Seoul, Korea) with a 14L-2 MHz transducer was used throughout this study. The elasticity range was set to 200 kPa for the patellar tendon and 300 kPa for the Achilles tendon in HQ-Vision mode with a persistence of 50%. All examinations were performed by an experienced sports medicine-trained clinician with a registered musculoskeletal sonography (RMSK) certification to eliminate inter-observer reliability.

**Figure 1:**
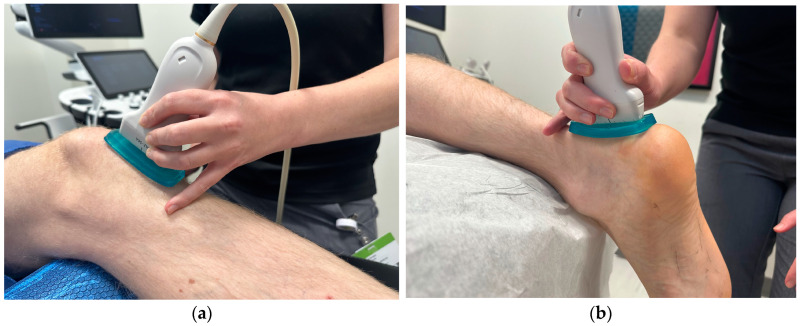
Patient positioning for image acquisition of the (**a**) patellar tendon at 30 degrees of knee flexion and (**b**) Achilles tendon in the prone position, with the foot relaxed, overhanging the bed.

**Figure 2:**
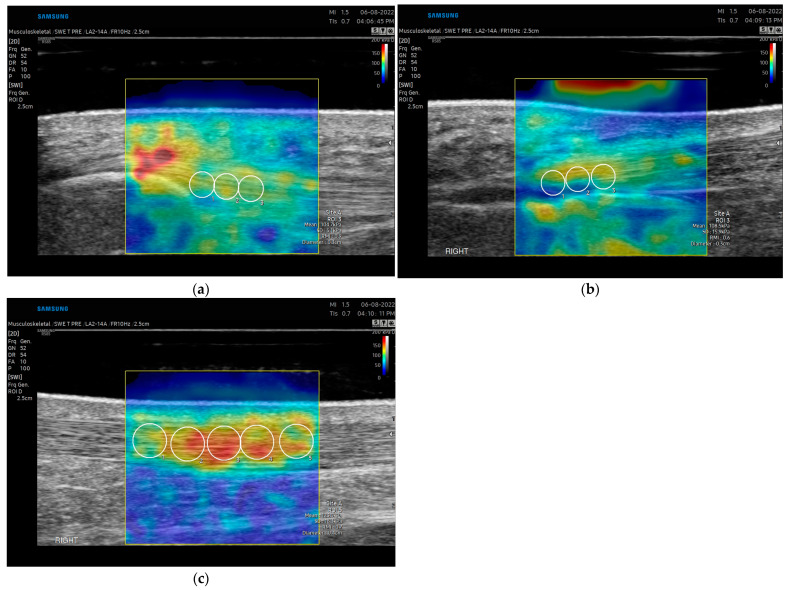
Longitudinal elastogram of (**a**) proximal patellar tendon, (**b**) insertional Achilles, and (**c**) midportion Achilles tendon in a 39-year-old healthy male. The Q-box is represented by the circles placed within the elastogram. The Q-box within the region of interest for the patellar tendon and insertional Achilles is 3 mm and the midportion 5 mm.

Image acquisition: For the patellar tendon, participants were assessed supine with the knee flexed and supported at 30 degrees with the hip in a neutral position (Figure 1a). Approximately 5 mm of ultrasound gel was applied using a gel standoff pad, and images were taken without significant pressure, given that pressure directly affects SWE measures [8]. The patellar tendon was first identified short axis and the transducer was then rotated 90 degrees into the long-axis position at the middle portion of the tendon. Careful observation was performed to avoid the medial or lateral aspect of the tendon. Five images were taken by the examiner to account for image artifact and reproducibility. The transducer was removed and again placed on the target tissue for each measurement. Data acquisition was performed at the proximal portion of the patellar tendon with initial measures at the peak of the lower apex of the patella. A 1.25 × 2.5 cm rectangular box was used as the region of interest. A 3 mm diameter q-Box was used for data analysis. Three adjacent measurements using the 3 mm q-box were taken spanning from the proximal to the midportion of the patellar tendon (Figure 2a). The first measurement (PT1) was placed immediately distal to the inferior border of the patella. Average values, not maximum values, were reported, as maximum values fluctuate with the ROI [8]. Images were only included if RMI (reliability of the measurement) was greater than 0.6 (determined by the machine output) and the IQR/Med (Interquartile/Median) ratio was under 30%. Outliers were removed if deemed an area of anisotropy based on the B-mode acquired image.

For the Achilles tendon, all participants were assessed in a prone position, with the knee fully extended and the foot relaxed, overhanging the bed (Figure 1b). The relaxed positioning of the tendon has been shown to have the highest reliability in assessment [9]. Approximately 5 mm of ultrasound gel was applied to maintain good contact between the ultrasound probe and skin without significant pressure using a gel standoff pad. The transducer was rotated in the long-axis position. Five images were taken by each examiner to account for image artifact and reproducibility. Data acquisition was performed at the insertion and midportion of the tendon (Figure 2b,c). The midportion of the Achilles tendon was determined by identifying the thickest portion of the tendon proximal to the insertion (approximately 5 cm from the calcaneus) and centering this midline with the transducer. A 1.25 × 2.5 cm rectangular box was used as the region of interest. A 3 mm and 4–5 diameter q-Box was used for data analysis of the insertional and midportion Achilles, respectively. Three measurements for the insertion and five measurements for the midportion were taken spanning the tendon and the length of the q-Box.

Image analysis: In biomechanics, stiffness refers to the relationship between stress and strain. In SWE, a longitudinal pulse is transmitted through tissue, causing displacement that is detected using pulse-echo ultrasound. This enables the measurement of shear wave velocity (V, in m/s). The shear wave velocity is directly related to the shear modulus (μ, in kPa) using the equation: μ = ρ × V^2^, where ρ represents tissue density (approximately 1000 kg/m^3^ in the human body). Hard tissues generally exhibit higher shear modulus and wave velocity compared to softer tissues, making this method useful for tendon evaluation [10]. Measurements of shear modulus are presented in kPa, velocity in m/s, and depth in cm. Initially, all images and data were reviewed and subsequently stored for further analysis.

### 2.4. Statistical Analysis

The intraclass correlation coefficient (ICC) was used as a measure of reliability agreement. For each study outcome, the ICC assesses the degree of agreement between multiple measurements taken on the same subjects, indicating how consistently the measurements agree with each other across repeated observations on the same individual; a higher ICC value signifies greater reliability, with a value close to 1 representing almost perfect agreement between repeated measurements, while a value closer to 0 indicates poor reliability. The ICC was estimated using variance components from a mixed effects linear model that included a random intercept for each subject that was used to calculate the between-subject variance and the residual variance as an estimate of the within-subject variance. The within-subject variance represents the variability in outcome measurements taken on the same subject, while the between-subject variance represents the variability between different subjects. The ICC is the ratio of the between-subject variance to the total variance (sum of the between-subject and within-subject variance). The ICC is large (i.e., ≈1) when there is little within-subject variance.

The repeatability coefficient (RC) indicates the maximum difference expected between two repeated measurements of the same outcome, with a 95% probability. Two readings by the same method (SWE) will be within 1.96√2s_w_ (or 2.77s_w_) for 95% of subjects. The within-subject standard deviation (s_w_) was estimated as the square root of the residual variance estimate from the mixed effects linear model. The RC was calculated by multiplying the within-subject standard deviation by the factor 2.77 (which is the square root of 2 multiplied by 1.96) [11]. The 2.77 factor is derived from the normal distribution, where 1.96 corresponds to the 95% confidence interval and the square root of 2 accounts for the fact that we are comparing two independent measurements. A smaller repeatability coefficient indicates better precision, meaning the measurements are more consistent when repeated. 

A linear regression analysis was performed to analyze the relationship between the patellar and Achilles tendon measurements (outcomes) with body mass index and sex as predictors. An adjusted mean was calculated for each patellar and Achilles tendon outcome. The adjusted mean and its 95% confidence interval for each outcome were defined as the predicted outcome value obtained by fitting the regression equation for males and for females at the mean body mass index using the same slope estimate for males and females. A one-sample *t*-test was used in linear regression to test the null hypothesis that the slope or the regression coefficient was equal to zero.

## 3. Results

Fifty-four healthy adults were recruited for this study and data were taken bilaterally for both the Achilles and patellar tendons of each participant. Detailed descriptive statistics are displayed in Table 1.

### 3.1. Patellar Tendon (PT)

**Figure 3:**
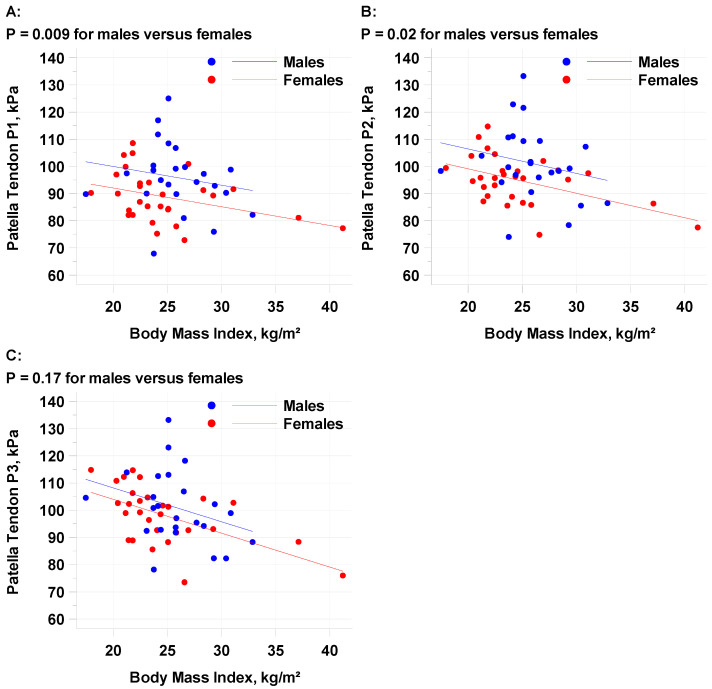
Linear regression analysis for patellar tendon stiffness with sex and BMI for each of the three measurement sites, (**A**) PT1, (**B**) PT2, and (**C**) PT3.

The mean SWE average of the patellar tendon locations (PT1-PT3, with PT1 representing the most proximal measurement point) for all subjects was 96.3 kPa (standard deviation 10.9 kPa) with moderate reliability (intraclass correlation coefficient, ICC = 0.50, 52, and 0.50 for PT1, PT2, and PT3). The repeatability coefficient of PT1 was 29.4 kPa, suggesting the differences between multiple measurements will be less than the RC in 95% of the cases made by a single observer. Males demonstrated a statistically higher kPa than females (Table 2) at positions PT1 (*p* = 0.009) and PT2 (*p* = 0.02), but not PT3 (*p* = 0.17). A higher BMI was correlated with a decrease in patellar tendon kPa (Figure 3) at all points. The mean PT1 decline per 1 kg/m^2^ increase in BMI was −0.6894 and the decline per 5 kg/m^2^ increase in BMI was −3.447. There was a trend towards a higher kPa in patients aged ≤29.5 (98.6 kPa versus 93.8 kPa). TAS greater than 5 had a higher kPa than lower TAS levels, but the number of individuals at each level was not enough to make statistical conclusions.

### 3.2. Achilles Tendon (AT)

**Figure 4:**
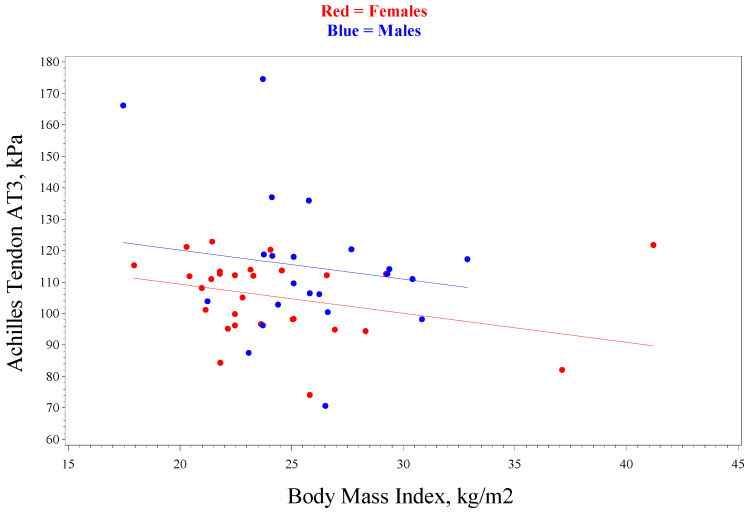
Linear regression analysis for Achilles tendon stiffness with sex and BMI for measurement of the insertional Achilles at measurement site 3 (AT3). *p* = 0.12 for testing slope = 0 and *p* = 0.04 for males versus females.

The mean SWE average of the insertional Achilles (AT1-AT3) for all subjects was 101.2 kPa (standard deviation 16.2 kPa) with moderate reliability (intraclass correlation coefficient, ICC = 0.57, 0.54, and 0.58 for AT1, AT2, and AT3). The repeatability coefficient (RC) of insertional Achilles AT1 was 40.4 kPa, suggesting the differences between multiple measurements will be less than the RC in 95% of the cases made by a single observer. The mean SWE average of the midpoint Achilles (AT1-AT3) for all subjects was 145.6 kPa (standard deviation 18.8 kPa) with poor reliability (intraclass correlation coefficient, ICC = 0.47, 0.40, 0.42, 0.43, and 0.40 for ATMP1–APMP5), but with moderate reliability for the left and right midpoint data. The repeatability coefficient of Achilles ATMP1 left was 47.9 kPa, suggesting the differences between multiple measurements will be less than the RC in 95% of the cases made by a single observer. The insertional AT measurements were higher in males compared to females (Table 3) but were only statistically significant for AT3 (*p* = 0.04). There was a decrease in kPa for insertional Achilles measurements in those with a higher BMI (Figure 4), but this did not reach statistical significance (*p* = 0.12). There was no difference in sex or BMI observed in the midportion of the Achilles. There was no trend observed with TAS scores for the insertional or midportion Achilles.

## 4. Discussion

This cross-sectional study aimed to establish baseline data on the mechanical properties of the patellar and Achilles tendons using ultrasound shear wave elastography. Our findings provide valuable insights into the variability of tendon stiffness in healthy individuals and highlight clinical considerations that may guide future research and clinical practice.

The results of this study confirm that shear wave elastography is a feasible method for assessing tendon stiffness in both the patellar and Achilles tendons. The highest agreement, as measured by the intra-class correlation coefficient (ICC = 0.5 to 0.7), was observed for the patellar tendon and the insertional Achilles tendon. In contrast, the midpoint Achilles tendon showed slightly lower agreement (ICC = 0.4 to 0.65), indicating moderate reliability. This study also found that factors such as BMI and sex significantly influence tendon stiffness, with higher BMI linked to lower stiffness in both tendons and males exhibiting greater stiffness than females. Additionally, younger participants generally showed greater tendon stiffness, suggesting that further research with larger cohorts is necessary to confirm these findings.

The study’s findings regarding tendon stiffness at specific locations—proximal, midportion, and distal sites of the patellar tendon, and insertional vs. midportion sites of the Achilles tendon—provide valuable insights into how tendon stiffness varies along the tendon’s length. Differences in stiffness at these locations may reflect regional variations in collagen alignment and tendon maturation, which could have implications for injury prevention and rehabilitation.

Increased BMI has been associated with lower tendon stiffness in this study, which may be explained by several factors. Adiposity around the tendon could alter the local mechanical environment, potentially leading to reduced stiffness. Furthermore, excess body weight may place additional strain on tendons, causing adaptive changes that could reduce their elastic properties. In addition, adiposity is associated with alteration of collagen fibers, impaired remodeling, and a chronic state of low grade inflammation which can affect tendon homeostasis [12,13]. These findings align with previous research, suggesting that higher BMI may reduce tendon stiffness and alter tendon structure [14].

Our findings are consistent with previous studies on the patellar tendon [14,15], but there is conflicting evidence regarding the Achilles. Wakker et al. found no significant effects of age, sex, or BMI on Achilles tendon stiffness, whereas Ruan et al. reported a decrease in elasticity with increasing age [16,17]. This discrepancy highlights the need for further research to better understand these relationships. In terms of activity level, we observed higher stiffness in the patellar tendon with greater activity, but no such trend was found for the Achilles tendon. Although our sample size was insufficient to draw definitive conclusions, previous studies have noted that higher activity levels tend to be associated with lower stiffness in the patellar tendon and higher stiffness in the Achilles tendon [18,19].

Clinically, SWE offers significant potential to enhance diagnostic capabilities, particularly in assessing tendon health. While B-mode ultrasound remains the standard diagnostic tool, it cannot directly measure tendon mechanical properties. In contrast, the accuracy of SWE has been well-documented. For example, Yurdaışık et al. assessed the accuracy of point shear wave elastography and two-dimensional SWE in diagnosing patellar tendinopathy, finding strong agreement with MRI-based clinical scoring [20]. Similarly, Zhang et al. compared the morphology and elastic properties of patellar tendons in athletes with and without unilateral patellar tendinopathy, revealing that those with tendinopathy had a higher elastic modulus [21]. In the Achilles tendon, Gatz et al. investigated whether SWE could distinguish between symptomatic and asymptomatic sides in unilateral Achilles tendinopathy, showing that patients with symptomatic tendinopathy exhibited higher SWE values compared to both the asymptomatic side and healthy controls [22]. These findings suggest that SWE could be a more sensitive diagnostic tool for tendon pathology than B-mode ultrasound alone [23,24].

Furthermore, SWE holds promise as a biomarker for monitoring treatment response. Previous research has demonstrated that SWE can track changes in tendon stiffness over time, providing objective data on the effectiveness of interventions such as physical therapy, orthobiologic treatments, or surgical procedures [5,25,26]. For clinicians, this could be especially valuable in the management of chronic tendon injuries, where monitoring recovery and guiding treatment decisions based on changes in tendon stiffness could lead to better patient outcomes.

This study has several limitations that should be considered when interpreting the results. First, the involvement of a single observer limits the ability to assess the consistency of SWE measurements across different practitioners. Interobserver and intra-observer reliability are important factors for the broader adoption of SWE in clinical practice. Future research should aim to establish standardized protocols for SWE imaging that include multiple observers, allowing for a more thorough evaluation of measurement variability.

Another limitation is the relatively small sample size, which restricts the generalizability of our findings. Despite this, the data on tendon stiffness variability, both within and between subjects, provide a valuable foundation for future studies and can help inform the design of larger trials.

Additionally, age-related differences in tendon stiffness and structure may have contributed to the observed variability. While a trend toward higher stiffness in younger participants was noted, the effect of aging on tendon properties remains not fully understood, and further research is needed to clarify this relationship. Moreover, although participants with visible tendon pathology were excluded through B-mode ultrasound at baseline, the presence of undiagnosed tendinopathy or subclinical tendon conditions could have influenced the results. Future studies should implement more comprehensive screening processes and consider the potential impact of subclinical tendon changes on stiffness measurements.

### Practical Implications

From a clinical standpoint, the findings from this study suggest that it is feasible to integrate SWE into routine musculoskeletal assessments, particularly for athletes and active individuals at higher risk for tendon injuries. There is the possibility that clinicians could use SWE to identify early changes in tendon stiffness that may precede visible structural damage on B-mode imaging. This could allow for earlier intervention and more personalized treatment strategies.

In the context of treatment monitoring, SWE could become an invaluable tool for tracking recovery in patients undergoing interventions for tendon injuries. By measuring changes in tendon stiffness over time, clinicians can gain objective data on the effectiveness of treatments, which could guide decisions on whether to progress or modify a rehabilitation protocol. SWE could also play a role in assessing the outcomes of regenerative medicine treatments, where changes in tendon mechanical properties may be key indicators of healing.

As this technology becomes more accessible and its reliability improves, it may also have a role in pre-participation assessments for athletes or individuals returning to sport after a tendon injury. By providing a clearer picture of tendon health, SWE could help prevent reinjury and optimize recovery protocols. However, widespread clinical adoption will require further validation, particularly in larger, more diverse populations, as well as the development of standardized protocols to ensure reproducibility and consistency across different settings and operators.

## 5. Conclusions

This pilot study provides valuable baseline data on tendon stiffness measurement via SWE in the patellar and Achilles tendons in healthy individuals. While the results contribute to understanding tendon properties, this study’s primary objective was to explore the feasibility of SWE as a tool for tendon assessment. Further research with larger cohorts is necessary to validate these findings and refine SWE protocols for clinical use, especially in diagnosing tendinopathies and monitoring treatment responses.

## Figures and Tables

**Table 1:** Descriptive statistics for patellar tendons and Achilles tendons (insertional and midpoint locations) measured by shear wave elastography (kPa).

	Patellar Tendons			Achilles Tendons, Insertional			Achilles Tendons, Midpoint		
Group	Sample Size	Mean (kPa)	Standard Deviation	Sample Size	Mean (kPa)	Standard Deviation	Sample Size	Mean (kPa)	Standard Deviation
All Subjects	54	96.3	10.9	52	101.7	16.2	52	145.6	18.8
Sex									
Male	24	99.3	12.4	22	105.9	20.4	22	142.6	18.3
Female	30	93.8	8.9	30	98.6	11.6	30	147.7	19.3
Age, median = 29.5									
≤29.5	28	98.6	11.4	28	100.0	15.7	28	141.7	20.2
>29.5	26	93.8	9.9	24	103.6	16.9	24	150.0	16.4
BMI, median = 24.4									
≤24.4	27	97.6	9.7	26	104.3	18.8	26	147.2	17.0
>24.4	26	95.2	12.1	25	99.1	13.1	25	143.7	21.2
Dominant Leg									
Right	42	95.7	10.2	41	102.6	17.3	41	144.5	18.0
Left	11	99.3	14.0	10	98.5	11.6	10	149.7	23.2
TAS Score									
2	2	94.5	0.1	2	87.5	6.7	2	125.0	26.1
3	7	95.2	6.1	6	106.2	29.2	6	153.4	26.5
4	15	95.6	14.6	15	101.7	7.9	15	144.2	16.6
5	6	93.4	11.5	4	116.0	30.0	4	153.1	22.7
6	9	97.1	7.4	9	90.0	14.4	9	148.7	15.2
7	13	99.1	12.0	14	104.8	8.7	14	142.0	19.4

BMI—body mass index; TAS—Tegner Activity Scale.

**Table 2:** Linear regression of patellar tendon (PT) (kPa) regressed on BMI and sex.

Outcome	Sex	Sample Size	Slope ± SE	*p* Value	Adjusted Mean (95% CI)	Mean Difference (95% CI)	*p* Value
PT1	Males	24	−0.6894 ± 0.3427	0.05	96.4 (92.1, 100.7)	7.9 (2.0, 13.8)	0.009
	Females	29			88.5 (84.5, 92.4)		
PT2	Males	24	−0.8993 ± 0.3487	0.01	101.7 (97.3, 106.1)	7.3 (1.3,13.3)	0.02
	Females	29			94.4 (90.4, 98.4)		
PT3	Males	24	−1.2471 ± 0.3497	<0.001	101.7 (97.3, 106.1)	4.2 (−1.8, 10.2)	0.17
	Females	29			97.5 (93.5, 101.5)		

PT—patellar tendon; SE—standard error; CI—confidence interval; adjusted mean value is in kPa.

**Table 3:** Linear regression of AT (kPa) regressed on BMI and sex.

Outcome	Sex	Sample Size	Slope ± SE	*p* Value	Adjusted Mean (95% CI)	Mean Difference (95% CI)	*p* Value
AT1	Males	22	−0.2100 ±0.5866	0.72	95.4 (87.8, 103.0)	5.7 (−4.4, 15.9)	0.26
	Females	29			89.6 (83.0, 96.2)		
AT2	Males	22	−0.4433 ± 0.5735	0.44	108.0 (100.6, 115.4)	7.6 (−2.3, 17.5)	0.13
	Females	29			100.4 (93.9, 106.9)		
AT3	Males	22	−0.9236 ± 0.5844	0.12	115.6 (108.0, 123.2)	10.8 (0.7, 20.9)	0.04
	Females	29			104.8 (98.2, 111.4)		

AT—Achilles tendon; SE—standard error; CI—confidence interval; adjusted mean value is in kPa.

## Data Availability

The data presented in this study are available on request from the corresponding author. The data are not publicly available due to privacy.
